# Melatonin Promotes Heterotopic Ossification Through Regulation of Endothelial-Mesenchymal Transition in Injured Achilles Tendons in Rats

**DOI:** 10.3389/fcell.2021.629274

**Published:** 2021-02-11

**Authors:** Jie Zhang, Jiajun Tang, Jie Liu, Bo Yan, Bin Yan, Minjun Huang, Zhongmin Zhang, Liang Wang

**Affiliations:** ^1^Department of Orthopedics, The Third Affiliated Hospital, Southern Medical University, Guangzhou, China; ^2^Academy of Orthopedics, Guangzhou, China; ^3^Division of Spine Surgery, Department of Orthopedics, Nanfang Hospital, Southern Medical University, Guangzhou, China

**Keywords:** heterotopic ossification, melatonin, EndMT, osteogenesis, neuroendocrine

## Abstract

Although heterotopic ossification (HO) has been reported to be a common complication of the posttraumatic healing process, the underlying mechanism remains unknown. Endothelial-mesenchymal transition (EndMT) is known to play a role in HO, and our recent study observed that neuroendocrine signals can promote HO by modulating EndMT. Melatonin, a neuroendocrine hormone secreted mainly by the pineal gland, has been documented to perform its function in the skeletal system. This study aimed at describing the expression of melatonin during the formation of HO in rat models of Achilles tendon injury and to further investigate its role in regulating EndMT in HO. Histological staining revealed the expression of melatonin throughout the formation of heterotopic bone in injured Achilles tendons, and the serum melatonin levels were increased after the initial injury. Double immunofluorescence showed that the MT2 melatonin receptor was notably expressed at the sites of injury. Micro-CT showed the enhancement of heterotopic bone volume and calcified areas in rats treated with melatonin. Additionally, our data showed that melatonin induced EndMT in primary rat aortic endothelial cells (RAOECs), which acquired traits including migratory function, invasive function and EndMT and MSC marker gene and protein expression. Furthermore, our data exhibited that melatonin promoted the osteogenic differentiation of RAOECs undergoing EndMT *in vitro*. Importantly, inhibition of the melatonin-MT2 pathway by using the MT2 selective inhibitor 4-P-PDOT inhibited melatonin-induced EndMT and osteogenesis both *in vivo* and *in vitro*. In conclusion, these findings demonstrated that melatonin promoted HO through the regulation of EndMT in injured Achilles tendons in rats, and these findings might provide additional directions for the management of HO.

## Introduction

Heterotopic ossification (HO) is a pathological process within the formation of mature bony tissues in extraskeletal sites, including joints, skeletal muscles and surrounding tissues, such as fascia and tendons (Meyers et al., 2019). HO can be conceptualized as aberrant tissue repair and is a devastating complication that is common in the healing processes of traumatic injuries, such as fractures, total hip arthroplasties, severe burns and spinal cord injuries (Li and Tuan, 2020). Unfortunately, this pathological phenomenon is also observed in a rare genetic disorder known as fibrodysplasia ossificans progressiva (FOP), which is caused by a gain-of-function mutation in the bone morphogenetic protein (BMP) type I receptor ACVR1 (Shore et al., 2006). The effectiveness of conservative treatments, such as NSAIDs and radiotherapy, is limited, and correction of HO relies on invasive surgeries; however, surgeries may lead to the postoperative recurrence of HO. Thus, there is an urgent need to gain knowledge about the mechanisms underlying aberrant repair and to develop a novel preventive treatment for HO.

The mechanisms of HO formation at sites of injury remain largely unclear. Currently, it is believed that the development of ectopic bone ossification is closely associated with the abnormal differentiation of mesenchymal stem cells (MSCs) at injured sites. MSCs from injured sites differentiate into osteoblasts or chondrocytes instead of differentiating into muscle or tendon cells due to the modulation of various signals or cytokines (Loder et al., 2018). Previous studies have reported that tendon-derived stem cells (TDSCs) (Jiang et al., 2017), hematopoietic stem cells and myofibroblasts are considered candidates for potential osteogenic cell types (Zhang et al., 2016). In a recent study, vascular endothelial cells that undergo the endothelial-mesenchymal transition (EndMT) process were found to play a role in HO formation in injured Achilles tendons in rats (Zhang et al., 2019). EndMT begins when endothelial cells delaminate from the organized cell layer and invade the underlying tissue, causing the loss of endothelial-specific markers, including vascular endothelial cadherin (VE-cadherin), cluster of differentiation 31 (CD31), tyrosine kinase with immunoglobulin-like and EGF-like 1 (Tie-1), and the gain of mesenchymal-specific markers, such as vimentin, neural cadherin (N-cadherin) and fibroblast-specific protein 1 (FSP-1). Increasing evidence now demonstrates that, in addition to its known functions in the cardiovascular system and cancer progression (Potenta et al., 2008), EndMT also plays a role in skeletal repair events (Piera-Velazquez et al., 2011; Medici and Olsen, 2012). Many reports have suggested that under pathological conditions, vascular endothelial cells that undergo EndMT are able to acquire the differentiation potential of mesenchymal stem-like cells, and these endothelial-derived MSC-like cells contribute to HO formation by differentiating into chondrocytes and osteoblasts (Medici et al., 2010; Medici and Olsen, 2012; Zhang et al., 2016). Recently, in a rat model of Achilles tendon injury, we observed the conversion of vascular endothelial cells into MSC-like cells through BMP-4- or TGF-β2-induced EndMT (Zhang et al., 2019). Furthermore, these newly formed cells represented the dedifferentiation of endothelial cells into a stem cell phenotype, and cells of this phenotype can subsequently redifferentiate into osteoblasts and chondrocytes and contribute to HO formation. Although we confirmed the critical effect of EndMT on HO formation, illustrating the underlying regulatory mechanism of EndMT in HO formation remains challenging.

Current studies hold the view that the effect of neuroendocrine signals in regulating the abnormal differentiation of MSCs at injured sites is one of the vital factors that affects the pathogenesis of HO formation (Gugala et al., 2018; Huang et al., 2018). A recent study demonstrated that Neurotrophin-3, with its neuroendocrine characteristics, can modulate the EndMT process in HO formation in rats (Zhang et al., 2019), suggesting that neuroendocrine cytokines can participate in the regulation of EndMT. Melatonin (*N*-acetyl-5-methoxytryptamine) is a neuroendocrine hormone that is secreted mainly by the pineal gland in mammals and is also expressed in other tissues, such as the ovary, testis, gut, and placenta (Fan et al., 2020). Many studies have demonstrated that melatonin regulates a variety of physiological activities, including the circadian cycle, oxidative stress and neuroendocrine processes, either through melatonin receptor 1A (MT1)/melatonin receptor 1B (MT2) or by acting directly as an antioxidant in cells (Majidinia et al., 2018). Currently, studies have focused on the role of melatonin in the skeletal system and have shown that melatonin is a key factor in regulating the osteogenic differentiation of various MSCs. A previous study reported that melatonin is capable of promoting the osteogenic differentiation and mineralization of MC3T3-E1 cells under hypoxic conditions via the activation of PKD/p38 pathways (Jang-Ho Son et al., 2014). In addition, melatonin showed a capacity to enhance the osteoblastic differentiation of human mandibular bone or iliac bone cells *in vitro* and to increase the volume of newly formed cortical bone of mice femora (Satomura et al., 2007). Moreover, melatonin enhanced the BMP-4-induced osteogenesis and increased the expression of osteogenic markers, especially Osterix, which is an essential transcription factor for the differentiation of C2C12 cells from preosteoblasts into mature osteoblasts (Han et al., 2017). Furthermore, a recent study demonstrated that the serum levels of melatonin are increased in ankylosing spondylitis (AS) patients, especially in those presenting with spinal bone ossification (Li et al., 2020). These previous studies have described the function of melatonin in osteogenesis or bone formation; however, few reports have elucidated the relationship between melatonin and HO formation. Importantly, our previous study indicates the effect of neuroendocrine signals such as Neurotrophin-3 on HO formation via EndMT modulation. Therefore, this study, as an extension of our previous research, focuses on the ability of melatonin to promote HO formation by inducing vascular endothelial cells to undergo EndMT in rats with Achilles tendon injuries.

## Materials and Methods

### Rat Achilles Tendon Injury Model and Melatonin Intervention Treatment

To investigate the roles of melatonin in the formation of ectopic bone in Achilles tendon injuries, a rat model of Achilles tendon injury was used, as we previously described (Zhang et al., 2019). These experiments were approved by the Ethical Committee for Animal Research (IAC1907001). At 4, 8, and 12 weeks after the operation, some rats (*n* = 6/time point) were euthanized, and their limbs were collected for gene expression and histological analyses of melatonin. A control group was euthanized (*n* = 6) as well. Additionally, for the HO formation study, the remaining rats were randomly divided into another four groups: the normal control, HO control, melatonin, and melatonin+4-P-PDOT groups (*n* = 6/group). The rats received a daily intraperitoneal injection of melatonin (50 μg/kg) (R&D Systems, Tocris Bioscience, Cat. #3550) for 12 weeks, and 4-P-PDOT (1 mg/kg) (R&D Systems, Tocris Bioscience, Cat. #1034) was also intraperitoneally administered daily. All the rats in the HO control group were administered a saline vehicle weekly. Twelve weeks after the Achilles tenotomy, all the rats were euthanized, and their limbs were collected for further study.

### Analysis of Serum Melatonin Levels in Rats With Achilles Tendon Injuries

Blood samples from rats with Achilles tendon injuries were acquired and centrifuged (2,000 rpm; 10 min; 4°C). Plasma fractions were stored at −80°C. The serum concentration of melatonin was determined using the ELISA Kit for melatonin (Rat: Cloud-Clone Corp. CEA908Ge) according to the manufacturer’s instructions.

### Micro-CT Analysis

HO volume was measured *in vivo* by micro-CT scanning (μCT 80, Scanco Medical, Bruttisellen, Zürich, Switzerland) of limbs from the experimental and control groups. The specimens was made a mean 20-μm thick slice. 60 kV and 150 μA were set for scanning. 3D images were reconstructed by the Micro-CT system software package, and the HO volume was calculated.

### Histological Analysis and Melatonin Immunohistochemistry

Specimens were collected from the rats with Achilles tendon injuries in the experimental and control groups, and the specimens were fixed, decalcified, dehydrated and processed for paraffin embedding, and cut into 4 μm thick slices. To describe HO, HE (Sigma-Aldrich, St. Louis, MI, United States), Safranin O and Fast Green (Sigma-Aldrich) (SOFG) staining were performed. The percentages of the calcified areas in the series sections were quantified by ImageJ software. After deparaffinization and rehydration, the sections were treated with 200 mg/ml proteinase K (Sigma–Aldrich) for 15 min at 37°C to unmask the antigen. The sections for immunohistochemistry (IHC) analysis were treated with 3% hydrogen peroxide for 15 min and then blocked with 1% goat serum at room temperature for 1 h. Then, the sections were immunostained with primary antibodies against melatonin (1:100, Abcam, ab203346) for IHC.

### MT1 and MT2 Melatonin Receptor Immunofluorescence Analysis in Rat Achilles Tendons

To investigate the primary receptor through which melatonin exerts its biological effect during HO formation, MT1 (1:100, Abclonal, A13030), MT2 (1:100, Abcam, ab203346), CD31 (1:100, Abcam, ab24590), SOX9 (1:100, Abcam, ab185966), and OCN (1:100, Abcam, ab13420) antibodies were used for immunofluorescence (IF). After incubation at 4°C overnight, a species-matched Alexa Fluor 488-, Alexa Fluor 594- or HRP-labeled secondary antibody was used (1:500) at 37°C for 1 h. For IHC, DAB (ZSGB-Bio, Beijing, China) was used as the chromogen, and hematoxylin was used for counterstaining. For IF, the sections were stained with DAPI (Roche Applied Science, Indianapolis, IN, United States). All the samples were observed under a microscope.

### Rat Aortic Endothelial Cell Isolation and Identification

To examine the effect of melatonin on EndMT, rat aortic endothelial cells (RAOECs) were isolated from SD rats as previously described (Zhang et al., 2019). The intact aorta was excised, a 6-0 suture was fixed on one end of the aorta and the inner surface of the aorta was flipped to the outside by drawing the suture through the lumen of the blood vessel with a blunt no. 5 suture needle. The turnover aorta was then cultured in endothelial cell medium (ECM) (ScienCell, Carlsbad, CA, United States) at 37°C under 5% CO_2_. The medium was refreshed every other day. After 1 week, the adherent cells were observed under a microscope. The adherent cells were identified as endothelial cells by IF staining with CD31 and Tie-1 antibodies (1: 100, Abcam, ab27851). Third passage endothelial cells were collected for further researches.

### CCK-8 Proliferation Assay

RAOECs were seeded at 1,000 cells/well in 96-well plates and treated with melatonin (10 ng/ml), BMP-4 (100 ng/ml), and TGF-β2 (100 ng/ml), respectively, for 48 h. Then, the cell number was determined using a CCK-8 proliferation assay kit (CCK-8, Dojindo, Japan) according to the instructions. The number of viable cells in each well was measured at an absorbance wavelength of 450 nm.

### The Induction of EndMT and Osteogenesis of RAOECs

For EndMT induction, RAOECs were cultured within melatonin (10 ng/ml), BMP-4 (100 ng/ml), and TGF-β2 (100 ng/ml) (R&D Systems) for 2 weeks. For osteogenesis, RAOECs were then cultured in osteogenic medium within 50 μmol/L ascorbic acid, 0.1 μmol/L dexamethasone, and 10 mmol/L β-glycerol phosphate (Sigma–Aldrich) after EndMT induction for another 2 weeks. For the functional study of melatonin, MT2 melatonin receptor inhibitor 4-P-PDOT (25 ng/ml) was added after the treatment of melatonin in RAOECs.

### Scratch Assay and Transwell Migration/Invasion Assay

The migratory ability of RAOECs after undergoing EndMT was measured by scratch and transwell migration/invasion assays. Cells were plated and a scratch was made in the cell monolayer with a P200 pipette tip for the scratch assay. The cells were then incubated in 5% CO_2_ at 37°C and photographed for up to 48 h. Closure of the scratch area was analyzed by Image-Pro Plus 6.0, and the scratch healing rate was quantified by the percent change in the scratch area. For the transwell migration and invasion assay, we seeded cells in the upper chamber, and the lower chambers were filled with low-serum ECM medium. After 48 h, the non-migrated cells were removed, and the migrated and invasive cells on the lower side of the membrane were stained with crystal violet. Images of four random sections were captured.

### Flow Cytometry

RAOECs were stained in a FACS buffer composed of PBS, 2% FCS, and 2 mM EDTA on ice for 20 min with the following specific antibodies: CD90 (Abacm, ab225), CD44 (Abacm, ab157107), CD31 (R&D Systems, AF3628), and CD105 (Abacm, ab2529). Flow cytometry was conducted on a BD Fortessa apparatus using FACSDiva software (BD Biosciences).

### ALP and ARS Cell Staining

To examine the potential effect of melatonin on osteogenic calcification activity, RAOECs were washed with PBS and fixed with 4% paraformaldehyde for 30 min after osteogenesis induction. ALP staining to detect osteogenesis was performed with cells cultured in osteogenic medium for 14 days according to ALP kit (Sigma-Aldrich). For Alizarin red staining, the cells were cultured for 21 days and then stained with 2% Alizarin red S staining solution (pH 4.2; Sigma-Aldrich) for 30 min at 37°C to visualize matrix calcification in the culture medium.

### Quantitative RT-PCR Gene Expression Analyses

Quantitative RT-PCR assays were used to analyze the effect of the treatments on the expression of regulatory genes in Achilles tendons or the cells from the cell culture experiments described above. Total RNA was extracted with TRIzol reagent (Life Technologies, Grand Island, NY, United States). cDNA was synthesized with the TaKaRa PrimeScript RT Reagent Kit (Takara Biotechnology Co. Ltd., Shija, Japan). Quantitative RT-PCR was performed using the TaKaRa SYBR Premix Ex Taq II kit according to the instructions (Takara). The primers were purchased from Shanghai Biological Engineering, the sequences are provided in Supplementary Table 1. The relative gene expression was calculated using the 2^–ΔΔ*CT*^ method (Kenneth and Livak, 2001).

### Western Blot Analyses

Western blot assays were used to analyze the effect of the treatments on the expression of regulatory genes in cells from the cell culture experiments described above. RAOEC lysates were collected and prepared for western blot analysis. We incubated the blots with primary antibodies (1,000-fold dilution) against VE-cadherin (Abcam, ab205336), Tie-1 (Abcam, ab27851), CD31 (Abcam, ab24590), N-cadherin (Abcam, ab76011), FSP-1 (Abcam, ab68124), Vimentin (Abcam, ab92547), OSX (Abacm, ab209484), OPN (Abacm, ab8448), Runx2 (CST, 12556S), OCN (Abcam, ab13420), and GAPDH (ZSGB-Bio, China) overnight at 4°C, followed by incubation with an anti-IgG horseradish peroxidase-conjugated secondary antibody (1:4000) for 1 h. Chemiluminescence was detected using an enhanced chemiluminescence (ECL) system. GAPDH served as the loading control.

### Statistical Analysis

For comparisons of two groups, significance was calculated by 2-tailed Student’s *t*-test. For comparisons of more than two groups, significance was calculated by 1-way ANOVA using SPSS 20.0 software (SPSS Inc., Chicago, IL, United States). A *P*-value of <0.05 was considered to be statistically significant.

## Results

### Melatonin in HO Formation in Rats With Achilles Tendon Injuries

**FIGURE 1:**
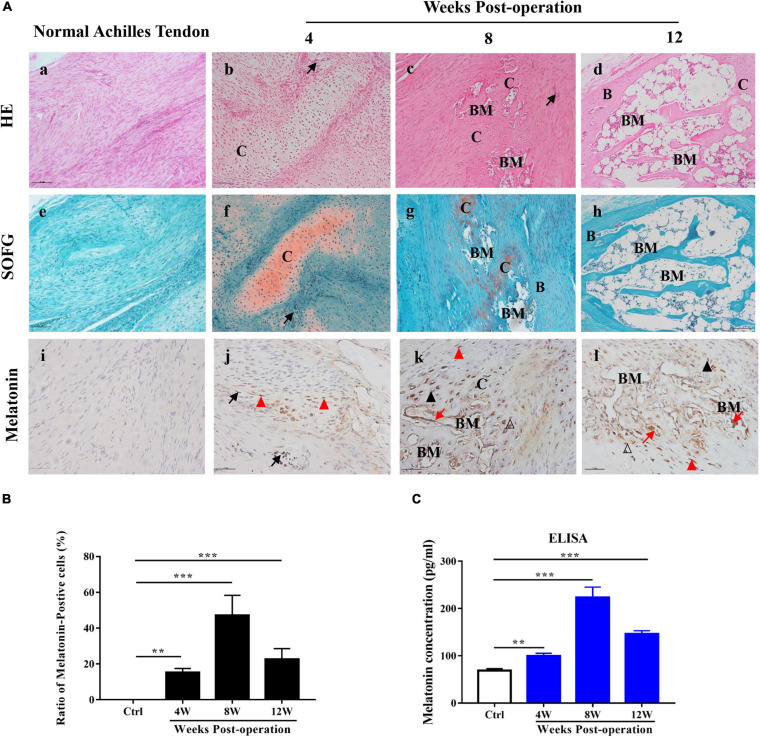
Melatonin in HO formation in rats with Achilles tendon injuries. **(A)** HE and SOFG staining of normal Achilles tendons **(a,e)** and HO specimens at 4, 8, and 12 weeks after the operation **(b–d,f–h)**. The occurrence of HO was seen at 8 weeks after the operation. Proteoglycan (red) and ectopic bone (green). Immunohistochemical staining of melatonin (brown) in normal Achilles tendons **(i)** and HO specimens at 4, 8, and 12 weeks after the operation **(j–l)**. IHC showed that melatonin was expressed in vascular endothelial cells, infiltrating mesenchymal cells, chondrocytes, osteoblasts and newly formed bone marrow cells. B, bone; BM, bone marrow; C, cartilage; Me, mesenchyme, black arrows point to vessel endothelial cells, red triangles point to infiltrating mesenchymal cells, black triangles point to chondrocytes, blank triangles point to osteoblasts and red arrows point to newly formed bone marrow cells. Scale bar, 10 μm. **(B)** The number of melatonin-positive cells in injured Achilles tendon specimens at the indicated time points. **(C)** Serum concentrations of melatonin from normal rats or rats with Achilles tendon injuries at different time points. The data represent the mean ± SD. **P < 0.01, ***P < 0.001 *versus* the normal control.

To determine the mechanism of HO pathogenesis, we examined surgical specimens from the rat model of Achilles tenotomy and identified these specimens histologically as being at an early stage of injury (4 weeks after the operation), osteogenesis stage (4–8 weeks after the operation) and mature stage (8–12 weeks after the operation). HE and SOFG staining of the HO specimens revealed cartilage layers adjacent to cancellous bone and bone marrow 8 weeks after the operation, while 12 weeks after the initial operation, large, well-developed cancellous bone and bone marrow were observed [Figures 1A(a–h)]. These histological staining results reveal a typical endochondral ossification process of HO formation in the injured Achilles tendons.

To investigate the role of melatonin in HO formation, we used an ELISA kit to examine the level of melatonin in the sera of the model rats at the indicated time points during HO formation, and normal rats were used as a control. Compared to the normal control rats, the model rats exhibited elevated serum levels of melatonin from 4 to 12 weeks, and the highest expression was observed 8 weeks after the operation (Figure 1C). Subsequently, to localize the expression of melatonin, we conducted IHC staining of melatonin during HO formation. Melatonin-positive cells were rarely found in the normal Achilles tendons [Figure 1A(i)], while in the injured Achilles tendons, melatonin-positive cells were observed from 4 to 12 weeks during ectopic bone formation [Figures 1A(j–l),B]; this result was consistent with the ELISA results. Additionally, melatonin was observed in vascular endothelial cells, in infiltrating mesenchymal cells at 4 weeks [Figure 1A(j)] and in chondrocytes, osteoblasts, and some newly formed bone marrow cells from 8 to 12 weeks [Figures 1A(k,l)]. These findings demonstrate that melatonin is important for HO formation and that melatonin might contribute to the different stages of HO formation in injured Achilles tendons.

### Roles of the MT1 and MT2 Melatonin Receptors Throughout HO Formation in Injured Achilles Tendons

**FIGURE 2:**
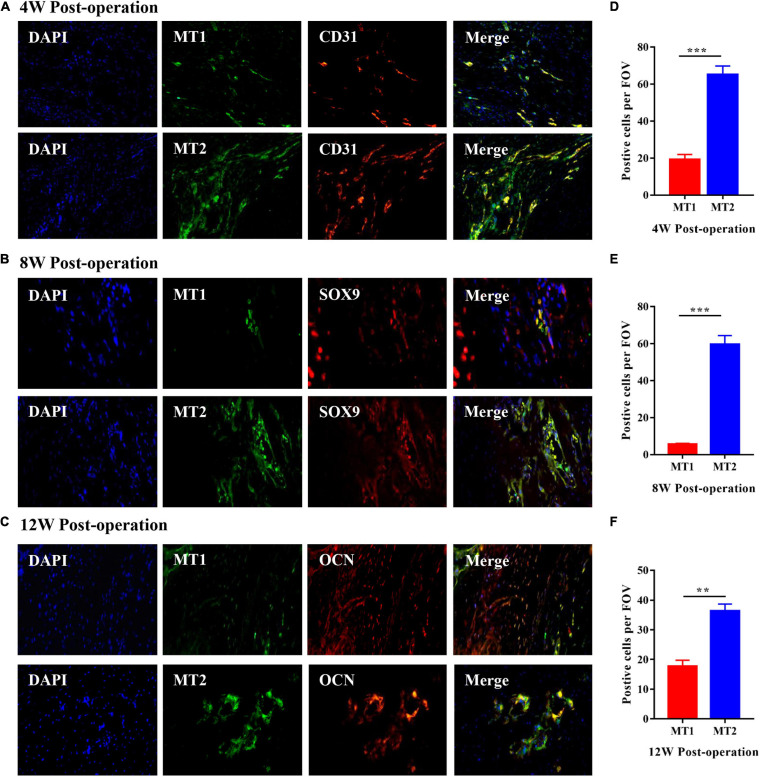
Roles of the MT1 and MT2 melatonin receptors throughout HO formation in injured Achilles tendons. **(A)** IFC analysis of HO specimens of injured Achilles tendons at 4 weeks after the operation with antibodies specific for the vascular endothelial marker CD31 (red), the MT1 melatonin receptor (green) and the MT2 melatonin receptor (green). Images show the coexpression of CD31 and MT1 (upper) and the coexpression of CD31 and MT2 (bottom). **(B)** IFC analysis of HO specimens from injured Achilles tendons at 8 weeks after the operation with antibodies specific for the chondrogenic marker SOX9 (red), the MT1 melatonin receptor (green) and the MT2 melatonin receptor (green). Images show the coexpression of SOX9 and MT1 (upper) and the coexpression of SOX9 and MT2 (bottom). **(C)** IFC analysis of HO specimens from injured Achilles tendons at 12 weeks after the operation with antibodies specific for the osteogenic marker OCN (red), the MT1 melatonin receptor (green) and the MT2 melatonin receptor (green). Images show the coexpression of OCN and MT1 (upper) and the coexpression of OCN and MT2 (bottom). Blue represents nuclear DAPI staining. Scale bar, 20 μm. **(D–F)** The number of MT2-positive cells was higher than that of MT1-positive cells at the indicated time points. The data represent the mean ± SD. **P < 0.01, ***P < 0.001 *versus* the MT1 group.

Heterotopic ossification formation in injured Achilles tendons is a process of endochondral ossification. Consequently, we identified the different stages of HO formation by conducting IFC on specimens from experimental rats; we used a CD31 antibody to represent the angiogenesis stage, a SOX9 antibody to represent the chondrogenesis stage and an OCN antibody to represent the osteogenesis stage (Figures 2A–C). The results showed that high numbers of CD31-positive cells were observed 4 weeks after the operation, while SOX9- and OCN-positive cells were mainly observed 8 and 12 weeks after the operation in the injured Achilles tendon; these results were in line with the histological analyses shown in Figure 1A. To investigate the roles of the MT1 and MT2 melatonin receptors in HO formation in injured Achilles tendons, specific IFC antibodies against MT1 and MT2 were used to stain the different melatonin receptors in specimens from rats with Achilles tendon injuries at 4, 8, and 12 weeks after the operation (Figures 2A–C). The data revealed that MT2-positive cells were more prominent at every indicated time point after the operation than MT1-positive cells (Figures 2A–F), suggesting a primary effect of the MT2 melatonin receptor on HO formation. Furthermore, to illustrate the potential relationship between the MT1/MT2 melatonin receptors and vascular endothelial cells, chondrocytes and osteoblasts in the progression of HO formation in injured Achilles tendons, we then performed double-labeling IFC staining on specimens from rats with Achilles tendon injuries (Figures 2A–C). The data showed the colocalization of melatonin with the vascular endothelial marker CD31, the chondrogenic marker SOX9 and the osteogenic marker OCN near the injured Achilles tendons, suggesting that melatonin receptors play a role in endochondral ossification in the injured Achilles tendon. These data suggest that melatonin and the MT2 melatonin receptor participate in HO formation in injured Achilles tendons.

### Effect of the Melatonin-MT2 Pathway on HO Formation in Injured Achilles Tendons

**FIGURE 3:**
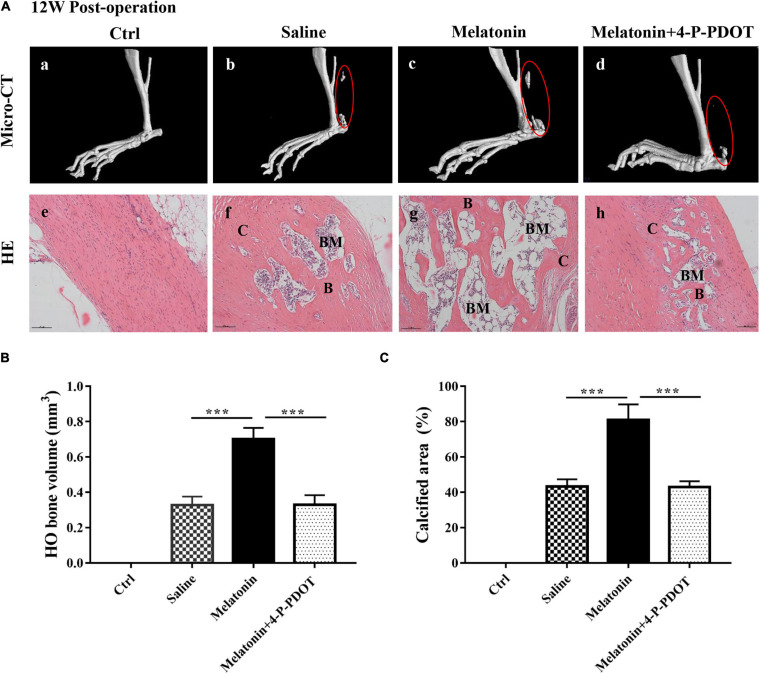
Effect of the melatonin-MT2 pathway on HO formation in injured Achilles tendons. **(A)** Micro-CT scans of HO formation in specimens from Achilles tendons from normal rats **(a)**, saline-treated rats **(b)**, melatonin-treated rats **(c)**, and melatonin- and 4-P-PDOT-treated rats **(d)** at 12 weeks after the operation. HE staining of normal and injured Achilles tendons and heterotopic ossification from the different groups mentioned above. B, bone; BM, bone marrow; C, cartilage, black arrows: blood vessels, black triangles: chondrocytes. The dashed line of the SOFG-stained sections shows the newly formed heterotopic bone. Scale bar, 100 μm. **(B)** HO volume (mm^3^) at injured Achilles tendons at 12 weeks. **(C)** Quantitative histological analyses of the proportions (%) of calcified areas at injured Achilles tendons at 12 weeks. The data represent the mean ± SD. ***P < 0.001 *versus* the saline control group or the melatonin group.

To evaluate the effect of the melatonin-MT2 pathway on HO formation *in vivo*, both melatonin and the MT2 receptor selective inhibitor 4-P-PDOT were administered to rats with Achilles tendon injuries, and we saline treatment and normal rats were used as controls. Micro–CT and HE staining were conducted to assess ectopic bone occurrence in rats with Achilles tendon injuries 12 weeks after surgery. The data show that compared with the normal control and saline treatments, the administration of melatonin to the rats with Achilles tendon injuries clearly increased the HO bone volume and calcified area, whereas melatonin combined with 4-P-PDOT treatment exerted a rescue effect on HO formation, showing a significant reduction in ectopic bone and calcified area (Figure 3A). In addition, quantitative analysis of the bone volume and calcified HO area suggested that compared with the normal control and saline treatment, melatonin significantly increased both the bone volume and calcified area of the injured Achilles tendons (Figures 3B,C). The results show that melatonin treatment significantly promotes HO formation, and inhibition of the melatonin-MT2 pathway suppresses HO formation in injured Achilles tendons.

### Role of Melatonin in EndMT of RAOECs

**FIGURE 4:**
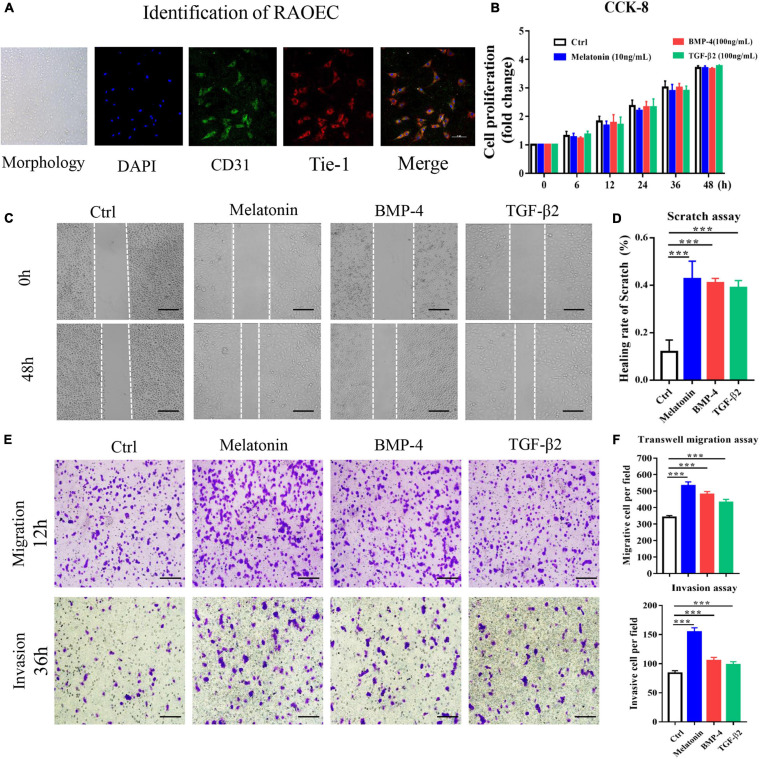
Melatonin induces EndMT in RAOECs. Primary RAOECs were cultured with melatonin, BMP-4 or TGF-β2 for 48 h. **(A)** The morphology of primary RAOECs and identification of primary RAOECs with CD31 and Tie-1 antibodies by IFC. Scale bar, 20 μm. **(B)** Cell viability was assessed by the CCK-8 assay. **(C)** Migratory function was evaluated by scratch, transwell and invasion migration assays **(E)** with quantitative analysis **(D,F)**. Images of the scratch, transwell and invasion migration assays, showing that melatonin increased RAOEC migration compared with the control treatment. Scale bar, 50 μm. The data represent the mean ± SD. ***P < 0.001 *versus* the control group.

As previously described, BMP-4 and TGF-β2 are identified as the inducer of EndMT (Zhang et al., 2019), which is associated with HO formation (Sun et al., 2016). Therefore, we induced EndMT in primary RAOECs by treatment with BMP-4 and TGF-β2 and investigated the effects of melatonin on endothelial- and mesenchymal-specific marker expression during EndMT. Initially, The morphology identification as well as IFC staining with CD31 and Tie-1 antibodies was carried out to identify the primary cells (Figure 4A). To evaluate the efficacy of melatonin treatment as well as to determine the suitable concentration, RAOECs were treated with 1, 10, 100, and 200ng/ml of melatonin as well as BMP-4 (100 ng/ml), and TGF-β2 (100 ng/ml), respectively, for 96 h in preliminary experiments (Supplementary Figure 1B). For the formal study, the CCK-8 assay showed that primary RAOECs exhibited no significant difference in cell proliferation after 48 h of culture in the presence of melatonin, BMP-4 and TGF-β2 (Figure 4B); these results suggest that stimulation with 10 ng/ml melatonin and 100 ng/ml BMP-4 and TGF-β2 did not cause toxic effects on the growth of RAOECs. We next performed scratch, transwell and invasion and migration assays to evaluate the effect of melatonin on cell motility. Compared with the control group, the melatonin-treated group exhibited notably enhanced scratch closure after 48 h of treatment, and the data showed that the cells in the BMP-4 and TGF-β2 treatment groups exhibited migration capacity similar to those in the melatonin treatment group (Figures 4C,D). Furthermore, we confirmed the effect of melatonin on the migration ability of RAOECs by conducting transwell and invasion assays. The data showed similar results to those of the scratch assay (Figures 4E,F).

**FIGURE 5:**
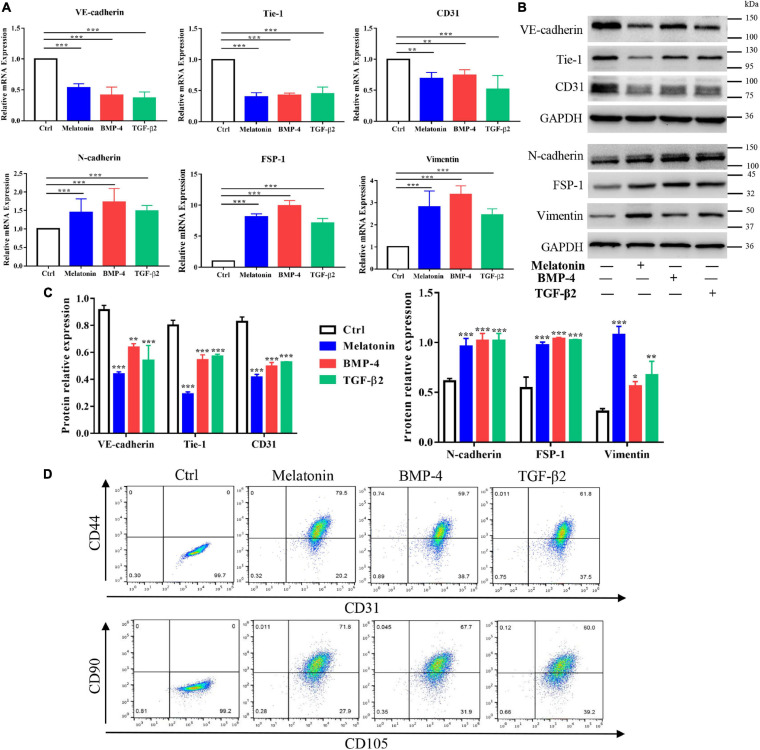
Role of melatonin in EndMT of RAOECs. Primary RAOECs were cultured with melatonin, BMP-4 or TGF-β2 for 2 weeks. **(A)** Total RNA was extracted for quantitative RT-PCR. Quantitative RT-PCR showed that melatonin downregulated the expression of the endothelial markers VE-cadherin, Tie-1, and CD31 and upregulated the expression of the mesenchymal markers N-cadherin, FSP-1 and vimentin. **(B,C)** Protein lysates were extracted for western blot and protein quantification analysis, and the data showed results similar to those of quantitative RT-PCR. **(D)** Flow cytometry results showed the co-expression of CD31 and CD44 as well as CD105 and CD90 in RAOECs cultured with melatonin, BMP-4 or TGF-β2. The data represent the mean ± SD. *P < 0.05, **P < 0.01, ***P < 0.001 *versus* the control group.

Subsequently, assessment of the expression of EndMT markers by quantitative RT-PCR and western blotting with protein quantification analysis suggested that treatment with melatonin, BMP-4 and TGF-β2 for 2 weeks downregulated the endothelial markers VE-cadherin, Tie-1 and CD31 and upregulated the mesenchymal markers N-cadherin, FSP-1 and vimentin at both the gene and protein levels (Figures 5A–C); these results indicated that melatonin can induce EndMT in RAOECs. To determine whether RAOECs acquire a stem cell–like phenotype after EndMT induction, flow cytometry was performed to examine the co-expression of the specific markers for endothelial cells (including CD31 and CD105) as well as mesenchymal cells (including CD44 and CD90). The data showed that the cells in control group were positive for CD31 and CD105 but negative for CD44 and CD90. Furthermore, compared to the control-treated cells, the melatonin-treated cells expressed not only the endothelial markers CD31 and CD105 but also the classic mesenchymal markers CD44 and CD90, and obvious expression of these markers was also observed in the BMP-4- and TGF-β2-treated cells (Figure 5D). Taken together, these data suggest that melatonin can induce EndMT and acquisition of a mesenchymal stem-like phenotype in EndMT-induced RAOECs.

### Effect of Melatonin on Osteogenesis in EndMT-Induced RAOECs

**FIGURE 6:**
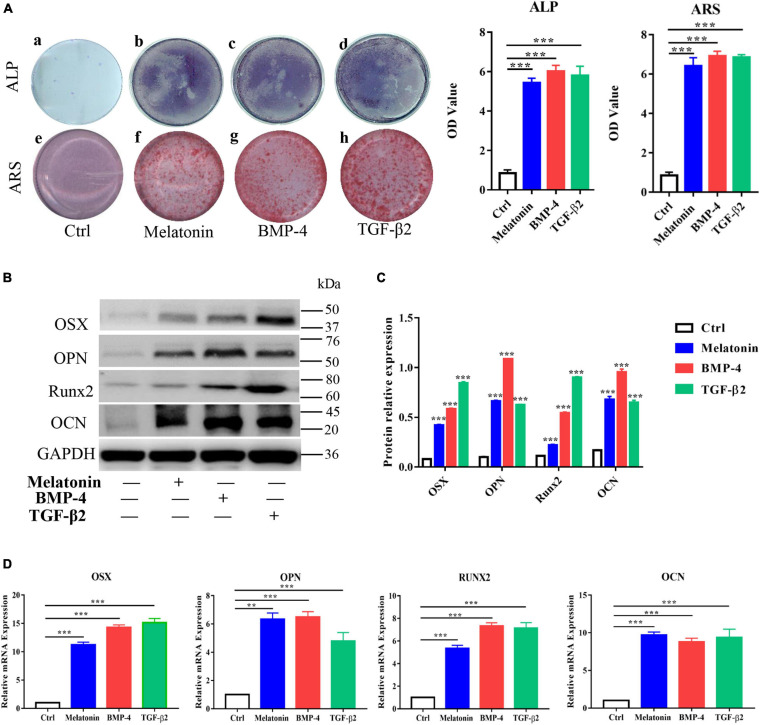
Effect of melatonin on osteogenesis of EndMT-induced RAOECs. Primary RAOECs were induced to undergo EndMT for 2 weeks and further induced to undergo osteogenic differentiation for another 3 weeks. **(A)** Cells in the experimental and control groups were stained with ALP and Alizarin Red. **(B)** Western blot with quantification analysis **(C,D)** quantitative RT-PCR both showed that melatonin enhanced the expression of the osteogenic markers OSX, OPN, Runx2, and OCN. The data represent the mean ± SD. **P < 0.01, ***P < 0.001 *versus* the control group.

To further investigate the effect of melatonin on HO formation, we next assessed the osteogenesis ability of melatonin-treated RAOECs *in vitro*. RAOECs were cultured with melatonin, BMP-4 and TGF-β2 for 2 weeks to induce EndMT, and then, these cells were induced to undergo osteogenic differentiation for another 3 weeks. As shown by cell staining, melatonin markedly increased ALP and Alizarin Red staining compared to the control treatment, and higher ALP and Alizarin Red staining absorbance was observed in the melatonin-treated cells. Similarly, BMP-4 and TGF-β2 led to a marked increase in ALP and Alizarin Red staining and absorbance (Figure 6A). Then, the osteogenic potential of the EndMT-induced RAOECs was confirmed by western blot with protein quantification analysis and quantitative RT-PCR, and the results showed an enhancement of osteogenesis-related genes and proteins, such as OSX, OPN, RUNX2, and OCN, in the melatonin treatment group (Figures 6B–D). Similar gene and protein expression patterns were observed in the BMP-4 and TGF-β2 treatment groups. In contrast, neither cell staining nor these osteogenic markers were detected in the control group. These results show that melatonin may contribute to the heterotopic bone formation associated with EndMT, confirming the osteogenic effect of melatonin on EndMT-induced RAOECs.

### Role of the Melatonin-MT2 Pathway in the Induction of EndMT

**FIGURE 7:**
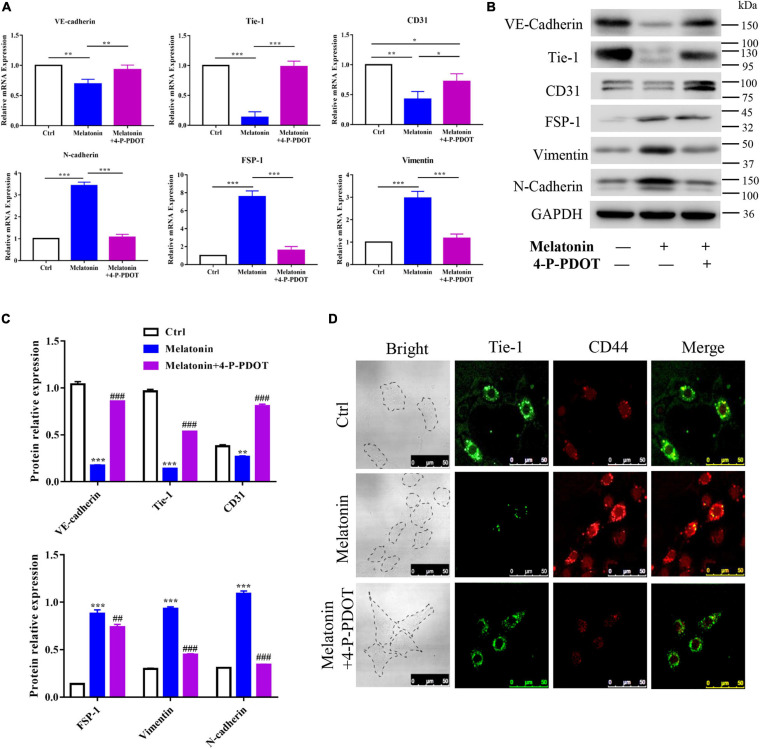
Role of the melatonin-MT2 pathway in the induction of EndMT. Primary RAOECs were induced to undergo EndMT with melatonin in the presence or absence of 4-P-PDOT for 2 weeks. **(A,B)** Quantitative RT-PCR and western blot with protein quantification **(C)** analyses showed that 4-P-PDOT reversed the melatonin-induced expression of endothelial markers and mesenchymal markers. **(D)** IFC staining also showed a reduction in the expression of MSC markers in the melatonin- and 4-P-PDOT-treated group. The data represent the mean ± SD. *P < 0.05, **P < 0.01, and ***P < 0.001 *versus* the control group and melatonin group. ^##^*P* < 0.01, ^###^*P* < 0.001 versus the melatonin group.

To explore the role of the melatonin-MT2 pathway in the induction of EndMT during HO formation, 4-P-PDOT, a selective inhibitor of the MT2 melatonin receptor, was utilized and the expression of EndMT markers in RAOECs was analyzed. The quantitative RT-PCR results showed that 4-P-PDOT rescued the effect of melatonin on EndMT in RAOECs, upregulating the endothelial-specific genes (VE-cadherin, Tie-1, and CD31) and downregulating the mesenchymal-specific genes (N-cadherin, FSP-1, and vimentin) induced by melatonin (Figure 7A). Similarly, western blot with protein quantification analysis demonstrated that 4-P-PDOT reversed the downregulation of endothelial-specific proteins and the upregulation of mesenchymal-specific proteins induced by melatonin (Figures 7B,C). In addition, IFC staining illustrated that 4-P-PDOT inhibited the melatonin-induced expression of the MSC marker CD44 (Figure 7D). Taken together, these results demonstrate that inhibition of the melatonin-MT2 pathway could suppress melatonin-induced EndMT in RAOECs.

### Inhibition of the Melatonin-MT2 Pathway Suppressed Osteogenesis in EndMT-Induced RAOECs

**FIGURE 8:**
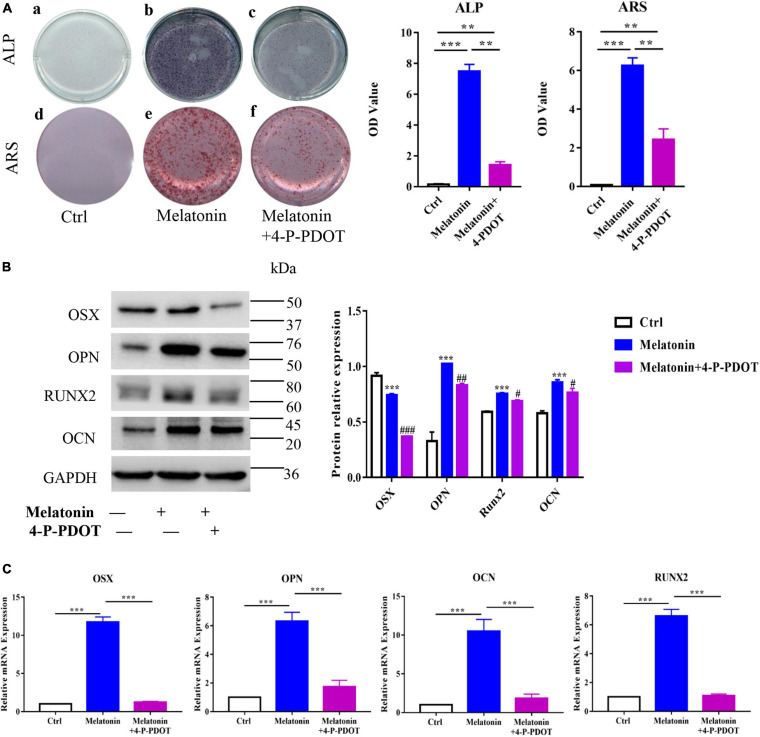
Inhibition of the melatonin-MT2 pathway suppressed the osteogenesis of EndMT-induced RAOECs. **(A)** The rescue effect of 4-P-PDOT on melatonin-induced osteogenesis was observed by ALP and Alizarin Red staining. **(B,C)** Western blot with protein quantification and quantitative RT-PCR showed that 4-P-PDOT reversed the melatonin-induced expression of osteogenic differentiation markers. The data represent the mean ± SD. **P < 0.01, ***P < 0.001 *versus* the control group and melatonin group. ^#^*P* < 0.05, ^##^*P* < 0.01, and ^###^*P* < 0.001 versus the melatonin group.

To further examine the effect of melatonin on osteogenesis in EndMT-induced RAOECs, the cells were treated with both melatonin and 4-P-PDOT. The cell staining data showed that the effect of melatonin in increasing ALP and Alizarin Red staining was inhibited by 4-P-PDOT, leading to a marked reduction in the ALP and Alizarin Red staining absorbance (Figure 8A). Moreover, to determine the role of 4-P-PDOT in melatonin-accelerated osteogenesis *in vitro*, the expression of osteogenic markers was evaluated by quantitative RT-PCR and western blot. The data demonstrated that 4-P-PDOT significantly decreased the melatonin-induced high expression of OSX, OPN, RUNX2, and OCN at both the gene and protein levels (Figures 8B,C). Taken together, these data suggest that the effect of melatonin on osteogenesis in EndMT-induced RAOECs can be inhibited by the MT2-specific inhibitor 4-P-PDOT, demonstrating that inhibition of the melatonin-MT2 pathway can suppress HO formation *in vitro*.

## Discussion

Although pathological analysis has revealed that heterotopic ossification is a process of endochondral bone formation, the underlying cellular and molecular events leading to HO formation remain unclear. Our previous study showed that endothelial-mesenchymal transition (EndMT) plays a role in HO formation in rats with Achilles tendon injuries (Zhang et al., 2019). Heterotopic bone specimens from rats with posttraumatic HO in their Achilles tendons showed the coexpression of the endothelial marker Tie-1 with the osteogenic differentiation marker OCN, suggesting that the osteoblasts in heterotopic lesions are of endothelial origin. In addition, our data showed that osteoblasts from normal bone tissue do not stain positive for endothelial markers, suggesting that the osteoblasts in HO are generated via EndMT. Furthermore, our previous study showed that neuroendocrine signaling factors, such as Neurotrophin-3, and other cytokines, such as BMP-4 and TGF-β2, induce EndMT in RAOECs by driving the transition from vascular endothelial cells into mesenchymal stem-like cells and promoting the gene and protein expression of EndMT and MSC markers. In the current study, the molecular mechanism by which melatonin participates in the EndMT during HO formation in rats with Achilles tendon injuries was investigated. First, we demonstrated the expression of melatonin on surgical specimens from a rat model of Achilles tenotomy-induced posttraumatic HO formation. Subsequently, we identified the role of the MT2 receptor in HO formation. Additionally, we illustrated that melatonin induced the EndMT of RAOECs and enhanced the osteogenic differentiation potential of MSC-like cells generated by EndMT. Furthermore, inhibition of the melatonin-MT2 pathway suppressed the role of melatonin in enhancing EndMT and osteogenic differentiation of EndMT-generated MSC-like cells.

Melatonin is a neuroendocrine molecule that is produced by the pineal gland and other organs and has been shown to exert a broad range of biological effects, including antioxidant, anti-inflammation, oncostatic, and circadian and endocrine rhythm regulatory effects (Hill et al., 2015; Mortezaee and Khanlarkhani, 2018; Ikegame et al., 2019). Recently, an increasing number of studies have reported that melatonin exerts a potent regulatory effect on the viability, proliferation and differentiation of various types of stem cells, such as MSCs, endothelial progenitor cells (EPCs) and induced pluripotent stem cells (iPSCs) (Luchetti et al., 2014). Due to its broad spectrum of effects, melatonin can regulate the differentiation of MSCs into osteogenic, chondrogenic and other lineages, opening up novel avenues for exploring its functions in the skeletal system. Previous studies have shown the effect of melatonin in promoting the osteoblastic mineralization and differentiation of MC3T3-E1 cells by increasing ALP activity and ALP, OSX, and OCN transcription levels in hypoxic environments, and the selective antagonist of melatonin receptors strikingly inhibits these effects (Jang-Ho Son et al., 2014; Son et al., 2017). Similarly, studies have shown that melatonin notably rescues the osteogenic differentiation of BMSCs by impairing the senescence of BMSCs caused by iron overload and preventing the reduction of cell proliferation (Yang et al., 2017a, b). Furthermore, enhancement in bone mass and facilitation of new bone growth were reported as positive functions of melatonin in the skeletal system (Majidinia et al., 2018). Although these previous studies investigated and reported the stimulatory effect of melatonin on osteogenic differentiation, the role of melatonin in aberrant bone tissue repair, especially in HO formation, remains largely unknown. In the present study, we observed that melatonin expression was markedly induced during the formation of heterotopic bone at the site of injured Achilles tendons. Melatonin was localized to vascular endothelial cells, chondrocytes, osteoblasts and some newly formed bone marrow cells at the injured sites. In addition, ELISA kits showed that melatonin expression in the serum was upregulated in a time-dependent manner at the early stage of osteogenesis and downregulated at the late stage of HO formation. These results illustrated that melatonin participates in HO formation in injured Achilles tendons in rats. While a previous study showed that higher serum levels of melatonin were observed in AS patients with spinal bone ossification formed (Li et al., 2020), our data showed that administration of melatonin led to increased heterotopic bone volume and calcified areas at the sites of injured Achilles tendons, suggesting that melatonin can accelerate HO formation. With these findings, we speculated that melatonin may play an important role in HO formation; however, the precise mechanisms and signaling pathways involved in this process remain unclear.

It has been suggested that the classic pathway through which melatonin exerts most its physiological effects is the receptor-dependent pathway (Luchetti et al., 2014). Melatonin receptor (MT), a member of the G protein-coupled receptor (GPCR) family, consists of two subtypes in mammals, MT1 and MT2 (Lacoste et al., 2015). Melatonin activates the coupled G protein by binding to the MT receptors, leading to the activation of many critical downstream signaling pathways. In addition to the central nervous system, MT receptors are also widely expressed in other tissues, including the skin, gonads and gastrointestinal tract (Stazi et al., 2020). Increasing evidence has shown that the MT2 melatonin receptor mediates the effect of melatonin on osteoblastic differentiation and bone mass regulation, while inactivation of MT1 does not affect bone mass (Sharan et al., 2017; Maria et al., 2018). Therefore, to investigate whether melatonin receptors were responsible for the effect of melatonin on HO formation in rats with Achilles tendon injuries, we measured the expression of the MT1 and MT2 melatonin receptors on surgical specimens from the experimental rats. Our data showed that melatonin receptors, including MT1 and MT2, were expressed at the sites of injured Achilles tendons throughout HO formation. Furthermore, we analyzed the expression of the MT1 and MT2 melatonin receptors over a time course and found that the MT2 melatonin receptor was more highly induced than the MT1 melatonin receptor at every stage of HO formation. These results supported the primary role of the MT2 melatonin receptor in HO formation in injured Achilles tendons, which was consistent with previous findings about the dominant effect of the MT2 melatonin receptor in bone. Additionally, our double-labeling immunostaining studies revealed the colocalization of the MT1 and MT2 melatonin receptors with the vascular endothelial marker CD31, the chondrogenic differentiation marker SOX9 and the osteogenic differentiation marker OCN in injured Achilles tendons during HO formation, suggesting that melatonin receptors, especially the MT2 melatonin receptor, mediate the effects of melatonin in modulating HO formation in rats with Achilles tendon injuries.

Previous studies have confirmed the role of EndMT in traumatic HO formation and showed the osteogenic differentiation ability of endothelial-derived MSC-like cells generated by EndMT (Lounev et al., 2009; Medici et al., 2010); however, none of these studies elucidated the underlying mechanisms of EndMT regulation. Neuroendocrine signals, which are regarded as potential mediators of EndMT (Salisbury et al., 2011; Lazard et al., 2015), have been shown to be associated with the bone system as they regulate the viability, proliferation and differentiation of osteoblasts (Takeda and Karsenty, 2008). Stimulatory actions of melatonin in the regulation of bone formation have been reported in various studies (Luchetti et al., 2014; Altındal and Gümüşderelioǧlu, 2016; Pytka et al., 2017). However, precise details revealing the regulatory mechanism of melatonin in EndMT during HO formation are lacking. In the current study our data showed that similar to BMP-4 and TGF-β2, melatonin induced the transition from vascular endothelial cells into mesenchymal cells by altering the cellular migratory and invasive functions. Melatonin induced the expression of mesenchymal cell markers and inhibited the expression of vascular endothelial cell markers. These data suggested that melatonin can induce EndMT. Furthermore, vascular endothelial cells that underwent melatonin-induced EndMT notably expressed MSC markers, such as CD44 and CD90, confirming that EndMT can generate mesenchymal stem-like cells. Subsequently, the present study showed that melatonin promoted the osteogenic differentiation of mesenchymal stem-like cells generated by EndMT by increasing the expression of OSX, RUNX2, and OCN at both the gene and protein levels; these data indicate that melatonin can promote HO formation by mediating EndMT. Taken together, the current study identified for the first time the profound role of melatonin in EndMT and HO formation.

To further illustrate the underlying signaling pathway by which melatonin participates in the HO formation associated with EndMT, we selected the MT2 melatonin receptor-specific inhibitor 4-P-PDOT for use in these studies. 4-P-PDOT, which has been shown to be an MT2-specific antagonist (Juszczak et al., 2014), was used to block the function of melatonin. As shown in our data, treatment with 4-P-PDOT not only suppressed the increase in heterotopic bone volume at injured Achilles tendons but also attenuated the effect of melatonin on inducing EndMT and promoting osteogenic differentiation. Treatment with 4-P-PDOT reversed the melatonin-induced EndMT by upregulating endothelial markers and downregulating mesenchymal and MSC markers at both the gene and protein levels in RAOECs *in vitro*. For osteogenesis, 4-P-PDOT reduced the melatonin-induced osteogenic differentiation by regulating gene and protein expression and inhibited mineralization in RAOECs. Additionally, 4-P-PDOT reversed the melatonin-induced acceleration of HO formation in rats with Achilles tendon injuries. Taken together, our data suggested an effect of the melatonin-MT2 pathway on HO formation both *in vivo* and *in vitro* and revealed that inhibition of the melatonin-MT2 pathway by 4-P-PDOT could suppress the induction of EndMT and HO formation in injured Achilles tendons. These findings may provide a novel preventive therapy for HO management.

Although we have identified a role of melatonin in EndMT on HO formation, in addition to vascular endothelial cells, melatonin has been reported to regulate the differentiation of a variety of MSCs at sites of injury. Therefore, further studies should be conducted to identify other candidate MSCs that are responsible for the formation of HO. Additionally, according to our data, the expression of melatonin was rarely found in normal Achilles tendons compared to injured Achilles tendons, suggesting that melatonin may not be generated and secreted near normal Achilles tendons. Thus, further research should focus on the potential sources of melatonin during HO formation at injured Achilles tendons. Moreover, the effects of melatonin on EndMT may be controversial according to a recent study (Liu et al., 2018). Liu et al. (2018) have demonstrated that melatonin could attenuate EndMT of GEnCs in diabetic nephropathy. Within this controversy, we may consider the different effects of melatonin on EndMT may be caused by the different biological pathway that melatonin plays its effects. Previous study has revealed that melatonin regulates various of physiological activities either through melatonin receptors (including MT1, MT2, or MT3) or by acting directly as an antioxidant in cells. The impairment of diabetic nephropathy was mainly related with oxidative stress injury, thus Liu et al. have suggested that melatonin’s antioxidant activity might be useful in treating diabetes and attenuating diabetic nephropathy, and reported that melatonin could attenuate EndMT of GEnCs. In addition, accumulating studies (Elbe et al., 2014; Onk et al., 2016; Winiarska et al., 2016) have supported the idea that melatonin has antioxidant and anti-inflammation activities in diabetic nephropathy. Based on these data and evidence, we believed that melatonin, which may play its direct antioxidant, attenuated EndMT of GEnCs in diabetic nephropathy. On the contrary, our data supported the primary role of the MT2 melatonin receptor in HO formation in injured Achilles tendons, which was consistent with previous findings about the dominant effect of the MT2 melatonin receptor in bone. Moreover, inhibition of melatonin-MT2 pathway both suppressed the EndMT induction and HO formation. Taken together, we believed that melatonin could promote EndMT in RAOECs through its MT2 melatonin receptor in HO formation of Achilles tendons injury rats. In addition, our main strength was that we utilized simple and repeatable rat models of Achilles tenotomy to illustrate the effects of melatonin on EndMT during HO, as well as the potential mechanisms. More importantly, we identified the role of the MT2 melatonin receptor in melatonin-induced EndMT and HO formation.

**FIGURE 9:**
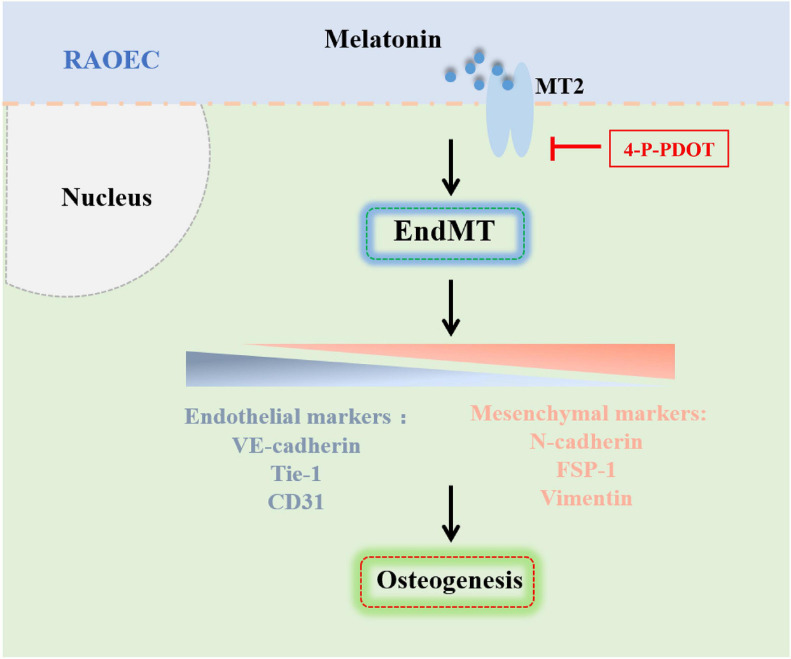
Schematic representation of the mechanism by which melatonin promotes HO formation by regulating EndMT through the MT2 receptor pathway. Melatonin promotes HO formation by inducing EndMT and generating vascular endothelial-derived MSC-like cells through the MT2 melatonin receptor pathway.

In summary, our analysis of rat models of Achilles tenotomy demonstrated that melatonin enhanced HO formation by promoting the osteogenic differentiation of vascular endothelial-derived MSC-like cells, which were generated by melatonin-induced EndMT. Inhibition of the melatonin-MT2 pathway could suppress EndMT induction and HO formation. Taken together, these findings illustrated the role and underlying mechanism of melatonin in the pathogenesis of HO formation (Figure 9), and these findings are expected to provide new strategies and targets for the clinical prevention and treatment of HO.

## Data Availability Statement

The original contributions generated for this study are included in the article/Supplementary Material, further inquiries can be directed to the corresponding author/s.

## Ethics Statement

These experiments were approved by the Ethical Committee for Animal Research (IAC1907001).

## Author Contributions

JZ, ZZ, and LW contributed to the study conception and design. Material preparation and data acquisition were performed by JL and JT. JZ, BiY, and BoY performed the experiments. JZ, MH, and LW contributed to the data analysis and interpretation. The first draft of the manuscript was written by JZ, ZZ, and LW. All authors approved the submitted version and agreed to be personally accountable for the authors own contributions and manuscript contents.

## Conflict of Interest

The authors declare that the research was conducted in the absence of any commercial or financial relationships that could be construed as a potential conflict of interest.
