# Supplementary figures and images for: Melatonin Promotes Heterotopic Ossification Through Regulation of Endothelial-Mesenchymal Transition in Injured Achilles Tendons in Rats
Source: Front Cell Dev Biol. 2021 Feb 11;9:629274. doi: 10.3389/fcell.2021.629274 (PMC7905064; doi:10.3389/fcell.2021.629274)

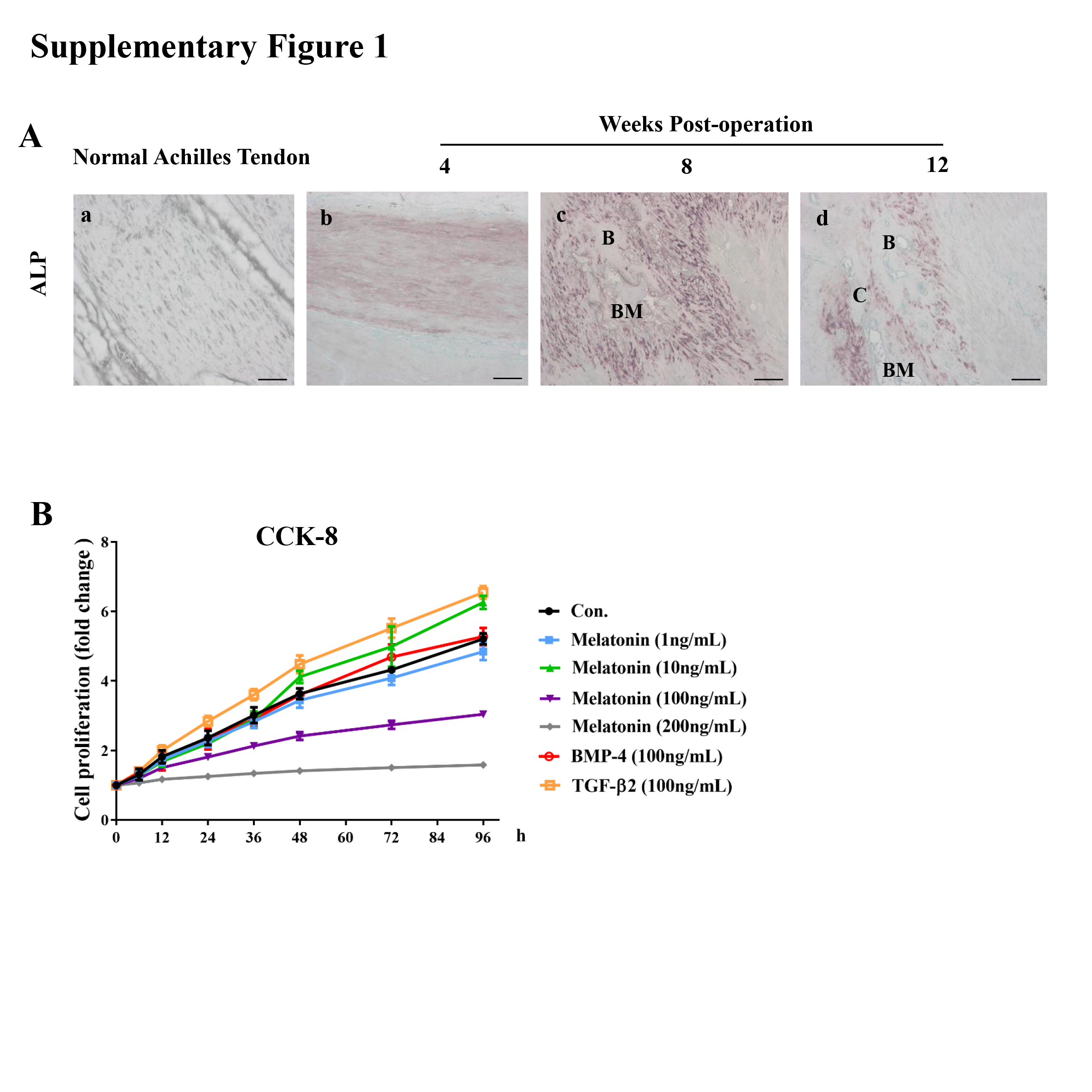

Supplement: Supplementary file 1 [file Image_1.TIF]

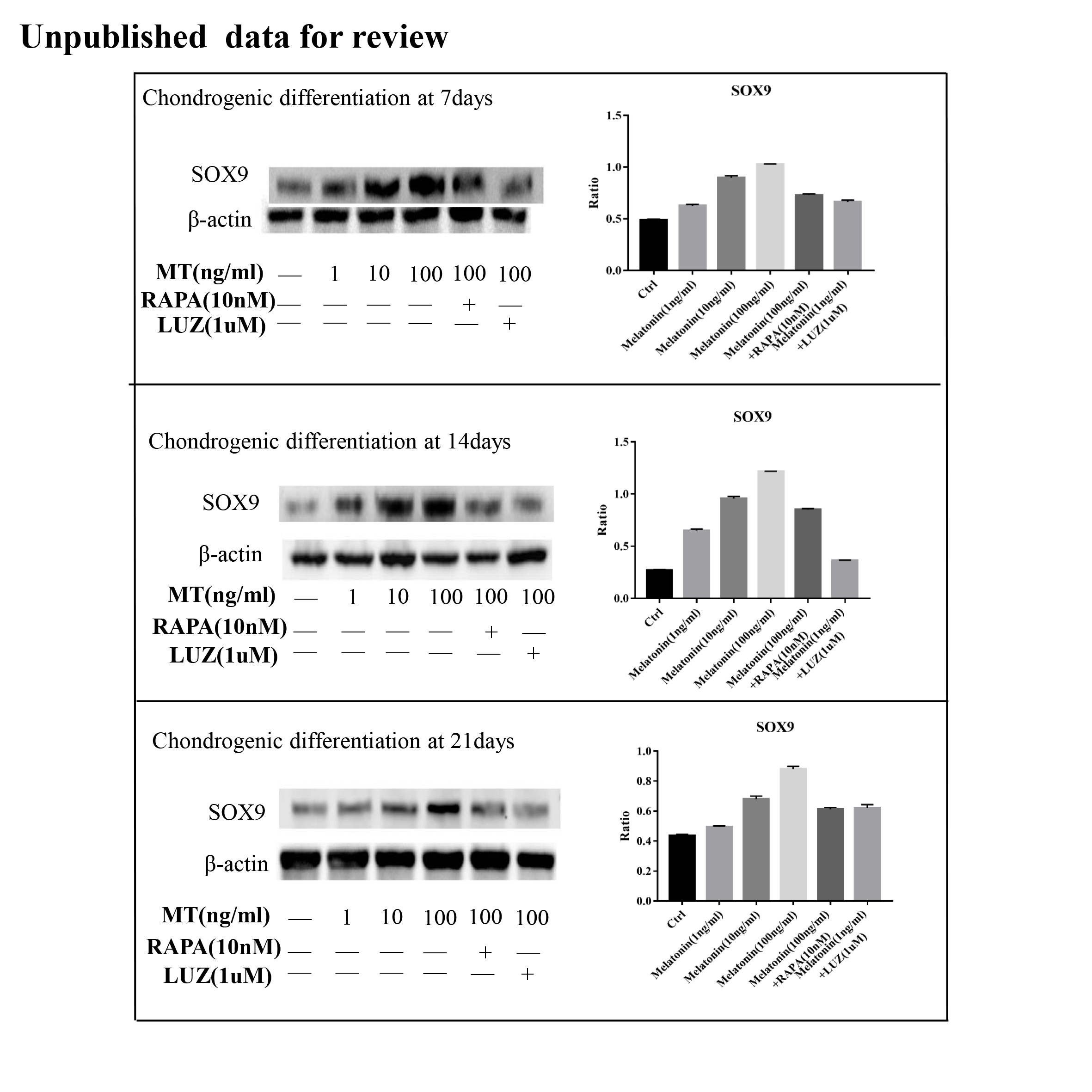

Supplement: Supplementary file 2 [file Image_2.TIF]

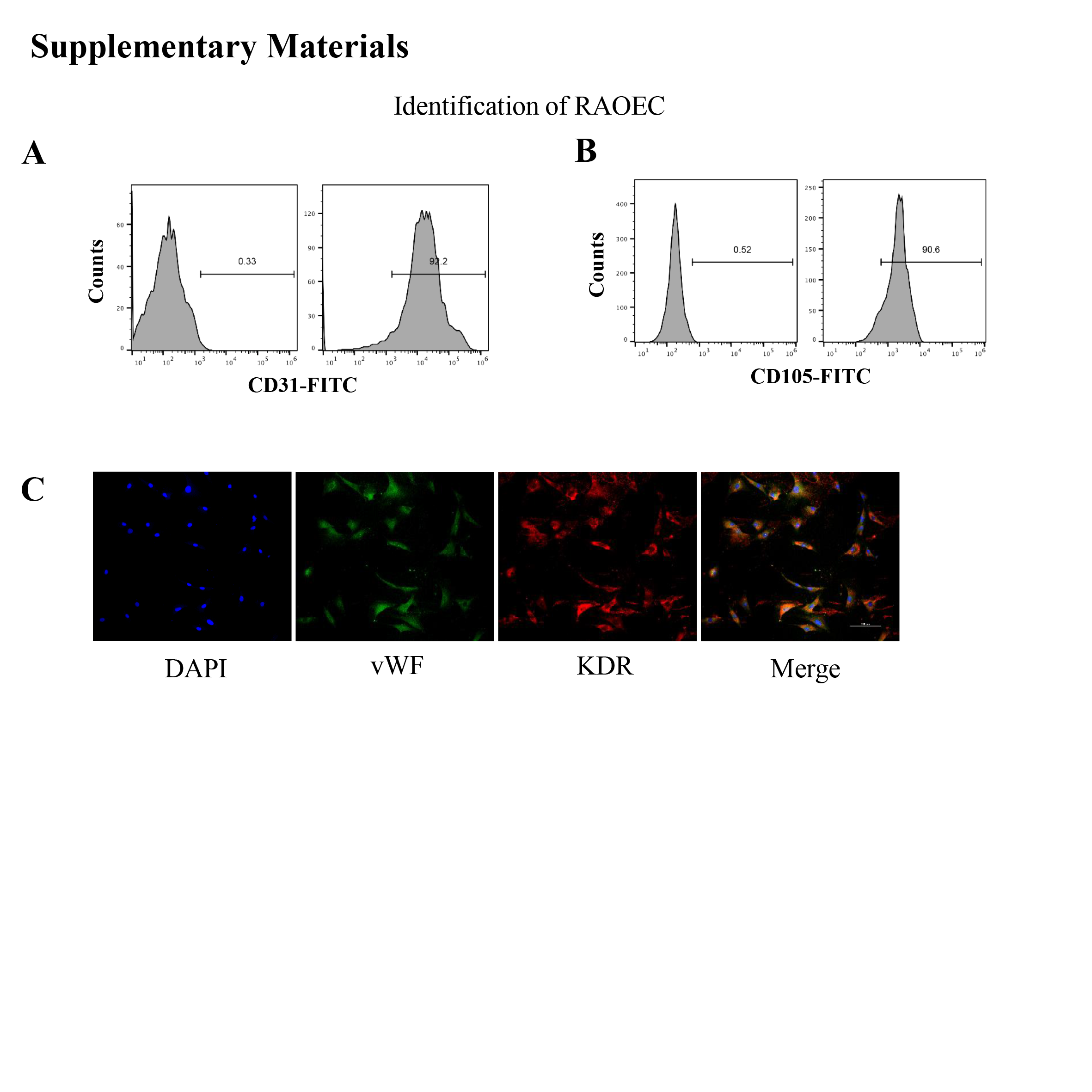

Supplement: Supplementary file 3 [file Image_3.TIF]
