# Supplementary material for: Melatonin Promotes Heterotopic Ossification Through Regulation of Endothelial-Mesenchymal Transition in Injured Achilles Tendons in Rats
Source: Front Cell Dev Biol. 2021 Feb 11;9:629274. doi: 10.3389/fcell.2021.629274 (PMC7905064; doi:10.3389/fcell.2021.629274)
Supplement: Supplementary file 4 [file Table_1.DOC]

**Table 1.** Primer sequences for quantitative RT-PCR

| **Gene** | **Forward primer (5’-3’)** | **Reverse primer (5’-3’)** |
| --- | --- | --- |
| GAPDH | GGCATTGCTCTCAATGACAA | TGTGAGGGAGATGCTCAGTC |
| VE-cadherin | ATCTTCAAGCCGTCCTGTGTG | TGAGGTTTGATCCGCATGATC |
| Tie-1 | AGAGCTGTCAGGAACAGTGC | GGAGTCGAGGTGCAGTCAAA |
| CD31 | CTTCACCATCCAGAAGGAAGA | CACTGGTATTCCATGTCTCTG |
| N-cadherin | CCCAGCGGTGGTTATGACTT | CGGCCACCATCTTGAGACTT |
| FSP-1 | TCTTGGTTTGATCCTGACTGCT | CCTGTTGCTGTCCAAGTTGC |
| α-SMA | TGGTGGAAACCCACAACGAA | ACACAGAGATCCGCAGTCCT |
| OSX | GCTTTTCTGTGGCAAGAGGTTC | CTGATGTTTGCTCAAGTGGTCG |
| OPN | TGAGAGCAATGAGCATTCCGAT | CAGGGAGTTTCCATGAAGCCA |
| RUNX2 | GTCGTCAGACCGAGAAGTGG | TCAAGTTCGAGGAAGCCGTG |
| OCN | CGCGTAAACGCCCTTTTGAT | AGTCTTGCAGCACCCGTAAA |
